# 34.2 IMPROVING THE DETECTION OF INDIVIDUALS AT RISK OF PSYCHOSIS IN SECONDARY MENTAL HEALTH CARE

**DOI:** 10.1093/schbul/sby014.143

**Published:** 2018-04-01

**Authors:** Paolo Fusar-poli, Grazia Rutigliano, Daniel Stahl, Cathy Davies, Ilaria Bonoldi, Thomas Reilly, Philip McGuire

**Affiliations:** Institute of Psychiatry, Psychology & Neuroscience, King’s College London

## Abstract

**Background:**

The overall effect of At Risk Mental State (ARMS) services for the detection of individuals who will develop psychosis in secondary mental health care is undetermined. The objective of the study presented in this lecture is to measure the proportion of individuals with a first episode of psychosis detected by ARMS services in secondary mental health services, and to develop and externally validate a practical web-based individualized risk calculator tool for the transdiagnostic prediction of psychosis in secondary mental health care.

**Methods:**

Clinical register-based cohort study. Patients were drawn from electronic, real-world, real-time clinical records relating to 2008 to 2015 routine secondary mental health care in the South London and the Maudsley National Health Service Foundation Trust. The study included all patients receiving a first index diagnosis of nonorganic and nonpsychotic mental disorder within the South London and the Maudsley National Health Service Foundation Trust in the period between January 1, 2008, and December 31, 2015. Data analysis began on September 1, 2016.

The main outcome is risk of development of nonorganic International Statistical Classification of Diseases and Related Health Problems, Tenth Revision psychotic disorders.

**Results:**

A total of 91 199 patients receiving a first index diagnosis of nonorganic and nonpsychotic mental disorder within South London and the Maudsley National Health Service Foundation Trust were included in the derivation (n=33 820) or external validation (n=54 716) data sets. The mean age was 32.97 years, 50.88% were men, and 61.05% were white race/ethnicity. The mean follow-up was 1588 days. The overall 6-year risk of psychosis in secondary mental health care was 3.02 (95% CI, 2.88–3.15), which is higher than the 6-year risk in the local general population (0.62). Compared with the ARMS designation, all of the International Statistical Classification of Diseases and Related Health Problems, Tenth Revision diagnoses showed a lower risk of psychosis, with the exception of bipolar mood disorders (similar risk) and brief psychotic episodes (higher risk). The ARMS designation accounted only for a small proportion of transitions to psychosis (n=52 of 1001; 5.19% in the derivation data set), indicating the need for transdiagnostic prediction of psychosis in secondary mental health care. A prognostic risk stratification model based on preselected variables, including index diagnosis, age, sex, age by sex, and race/ethnicity, was developed and externally validated, showing good performance and potential clinical usefulness.

**Discussion:**

This lecture will introduce a new online individualized risk calculator which can be of clinical usefulness for the transdiagnostic prediction of psychosis in secondary mental health care. The risk calculator can help to identify those patients at risk of developing psychosis who require an ARMS assessment and specialized care. The use of this calculator may eventually facilitate the implementation of an individualized provision of preventive focused interventions and improve outcomes of first episode psychosis.

